# Structural, Mechanical, and Barrier Properties of the Polyvinylidene Fluoride-Bacterial Nanocellulose-Based Hybrid Composite

**DOI:** 10.3390/polym16081033

**Published:** 2024-04-10

**Authors:** Aleksandra Janićijević, Suzana Filipović, Aleksandra Sknepnek, Ana Salević-Jelić, Radmila Jančić-Heinemann, Miloš Petrović, Ivan Petronijević, Marina Stamenović, Predrag Živković, Nebojša Potkonjak, Vladimir B. Pavlović

**Affiliations:** 1 The Academy of Applied Technical Studies Belgrade, 11000 Belgrade, Serbia; mstamenovic@atssb.edu.rs; 2 Institute of Technical Sciences of SASA, 11000 Belgrade, Serbia; suzana.filipovic@itn.sanu.ac.rs; 3 Faculty of Agriculture, University of Belgrade, 11000 Belgrade, Serbia; aleksandras@agrif.bg.ac.rs (A.S.); ana.salevic@agrif.bg.ac.rs (A.S.-J.); vladimirboskopavlovic@gmail.com (V.B.P.); 4 Faculty of Technology and Metallurgy, University of Belgrade, 11000 Belgrade, Serbia; radica@tmf.bg.ac.rs (R.J.-H.); mpetrovic@tmf.bg.ac.rs (M.P.); peca@tmf.bg.ac.rs (P.Ž.); 5 Faculty of Physics, University of Belgrade, 11000 Belgrade, Serbia; ivanpetronijevic@ff.bg.ac.rs; 6 Vinča Institute of Nuclear Sciences—Nation Institute of the Republic of Serbia, University of Belgrade, Mike Petrovica Alasa 12-14, 11000 Belgrade, Serbia

**Keywords:** laminate composite material, PVDF, BNC, tensile test, water vapor permeability

## Abstract

This study presents an analysis of films which consist of two layers; one layer is PVDF as the matrix, along with fillers BaTiO_3_ (BT), and the second is one bacterial nanocellulose (BNC) filled with Fe_3_O_4_. The mass fraction of BT in PVDF was 5%, and the samples were differentiated based on the duration of the mechanical activation of BT. This innovative PVDF laminate polymer with environmentally friendly fillers aligns with the concept of circular usage, resulting in a reduction in plastic content and potential improvement of the piezoelectric properties of the entire composite. This work presents new, multifunctional “green” packaging materials that potentially could be a good alternative to specific popular materials used for this purpose. The synthesis of the films was carried out using the hot press method. Tensile tests, water vapor permeability examination, and structural analyses using SEM-EDS and FTIR have been conducted. The sample PVDF/BT20/BNC/Fe_3_O_4_ exhibited the best barrier properties (impermeability to water vapor), while the highest tensile strength and toughness were exhibited by the PVDF/BT5/BNC/Fe_3_O_4_ sample.

## 1. Introduction

Contemporary demands for intelligent and smart packaging materials set high standards in terms of durability, product protection, multifunctionality, and environmental sustainability. Packaging needs to simultaneously provide a reliable barrier, extend the shelf life of the products, reduce the environmental footprint, and allow for an attractive design. In this context, composite materials become pivotal, and one of the prominent solutions is composites with poly(vinylidene fluoride) (PVDF) [1]. PVDF stands out as an exceptionally chemical- and heat-resistant versatile polymeric material, showcasing its adaptability across a diverse range of applications. It also possesses good oxidative stability, making it suitable for practical use in various fields, such as sensors, electronic devices, piezoelectric generators, tissue engineering scaffolds, and portable analytical devices [2,3]. Furthermore, PVDF is also environmentally friendly, featuring very low adhesion regarding microorganisms (growth of fungi, algae, and microbial films) [4,5]. On the other hand, it is one of the least flammable materials, melting slowly without emitting significant amounts of smoke [4,6,7]. Composites based on PVDF stand out in the field of packaging due to their low permeability to gases and liquids, making them ideal for the packaging of food, pharmaceuticals, and other goods where preserving freshness is crucial. Additionally, the extensive use of PVDF, especially in the sensor field, holds the potential for the development of new multifunctional packaging materials. This could significantly contribute to advancing product monitoring, providing additional security and information for consumers.

PVDF exhibits a complex structure with at least five crystalline phases (α-, β-, γ-, δ-, and ε-phases). Among these, the nonpolar α-phase and the polar β-phase are most commonly present [8]. It has been observed that, during PVDF production by conventional methods, melts and solutions tend to favor the formation of the nonpolar α-phase, while the desired electroactive polar β-phase can be achieved through methods such as mechanical stretching [9], rapid cooling [10], rapid thermal treatment, as well as the addition of various ceramic fillers [11]. The favoring of polar structures is responsible for the piezoelectric and pyroelectric properties of PVDF.

Fillers in the PVDF matrix can be effectively incorporated, and, beyond favoring the β-phase, they can enhance both barrier and mechanical properties [12].

In the process of developing intelligent materials for packaging, sensors, and nanogenerators, it is crucial to carefully combine fillers with the PVDF matrix. On the one hand, careful selection of synthesis methods and control over the synthesis process can contribute to favoring a specific phase of PVDF. Special attention has to be paid to the proportion of individual fillers, as uncontrolled incorporation of fillers can lead to a complete loss of flexibility or other mechanical properties, limiting their potential applications [13].

In the authors’ previous research, the latest and some of the most intriguing fillers were assessed for combining with PVDF [14,15,16]. It was found that an increase in cellulose content in polymer matrices noticeably improves the tensile strength of composite membranes [17,18]. Due to its renewable nature and biodegradability [19], cellulose is often impregnated into the polymer matrix of PVDF to enhance mechanical strength, as reported by studies [20,21]. The purest source of cellulose fibers, in addition to being the most environmentally and economically viable, is bacterial nanocellulose (BNC) [22].

As a highly efficient nucleating agent supporting the formation of the desired β-phase in PVDF, ceramic particles, specifically BaTiO_3_ (BT) powder, have proven to be effective. Interactions between BT and PVDF were explored, resulting in improved thermal stability and increased piezoelectricity [23,24,25].

Additionally, the impact of cellulose on PVDF/BT was reported to the authors at [21]. They demonstrated that the proposed composite PVDF/BT with cellulose (CNC) reinforced the PVDF film without compromising its flexibility and electrical properties. The process of obtaining PVDF/BT/CNC films was conducted using the solution casting method with dimethylformamide (DMF), and a plant-based cellulose source was used. This cellulose source represents an environmentally less sustainable solution compared to bacterial nanocellulose (BNC). Cellulose generated by bacteria does not require aggressive and high energy consuming processes of purification such as with plants. Other advantages of BNC include finer threads, improved crystallinity, greater mechanical strength, etc. In addition, the crystal structure of BNC is identical to that of plant cellulose [26]. Depending on the used species, cultivation conditions and duration, its degree of polymerization (DP) can be influenced. In the research of Semjonovs et al. [27], *Komagataeibacter rhaeticus* strain P1463 was favored for BNC production upon *Komagateibacter hansenii* strain B22 due to produced BNC with a higher DP that reached 2508 glucose units after 14 days of cultivation and 3300 glucose units after 40 days. *Acetobacter xylinum* after 4–10 days produced BNC with DP between 1150 and 2000 glucose units [28].

Barrier properties are very important in the development of intelligent packaging materials. Packaging materials must effectively shield products from external factors, and the permeation of water, gases, and vapors into plastic materials can have significant effect on their performance [29]. The study Keller et al. [30] has investigated various theoretical and experimental aspects of water and gas permeation in polymeric materials. Barrier characteristics prevent unwanted interactions between the packaging and the external environment, preserving the integrity of the product throughout the entire supply chain [31,32]. Precisely adjusting these properties, along with the PVDF matrix and the fillers, potentially enables the production of films, for various applications, that can not only provide optimal protection but also contribute to extending the shelf life of a specific product.

In parallel, understanding the mechanical characteristics of materials plays also very significant role in designing functional packaging, sensors, etc. Flexibility, strength, and elasticity determine how the material responds to physical forces during handling, transportation, and use [33], and achieving an optimal balance between rigidity and flexibility is a significant task. For example, in smart packaging with integrated sensors or nanogenerators, where precise interaction with the environment is required, knowledge of mechanical properties enables the design of materials resistant to damage while remaining flexible enough to adapt to various usage conditions [34]. Recent studies have reported improvements in the mechanical properties of PVDF composites with fillers, such as carbon fibers [35], graphene [36], graphene oxide-titania layers [37], which have shown an increase in tensile strength and the modulus of elasticity. There is significantly less literature available on PVDF composites with environmentally friendly fillers. Study [38] has demonstrated that cellulose nanofibers contribute to the reinforcement of the PVDF matrix, while [39] has claimed that the incorporation of BT particles into PVDF leads to increased stiffness and strength.

In the present research, the PVDF/BT was synthesized with a variety of times for the mechanical activation of BT with added BNC, previously modified with Fe_3_O_4_, to produce PVDF/BT/BNC/Fe_3_O_4_ composites. The purest source of cellulose fibers was introduced without the use of heavy chemicals, as well as without the use of DMF, as the composite was synthesized using the hot press method [16].

From the aforementioned studies, it can be concluded that they provided a considerable number of results reporting on the impact of fillers on PVDF concerning dielectric, piezoelectric, and electrical properties. Mechanical properties were addressed only in the study that was conducted by Ram et al. [21], especially in the case of complex systems with PVDF, while no results regarding barrier properties have been found in research on PVDF composites. Therefore, the study aimed to explore the impact of BT and BNC/Fe_3_O_4_ nanofillers on the mechanical and barrier properties of PVDF composite films. Furthermore, phase analysis and surface structures were examined using a scanning electron microscope (SEM-EDS) and Fourier transform infrared (FTIR) spectroscopy. The mechanical properties of the synthesized composite films were assessed by analyzing Young’s modulus (at 0.1–1% strain), toughness modulus, elongation at break, and ultimate strength. Additionally, the barrier properties were investigated by analyzing the permeability of water vapor through the laminated, layered composite samples.

## 2. Experimental

### 2.1. Materials

For the synthesis of bacterial nanocellulose (BNC), acetic acid bacteria (*Komagataeibacter rhaeticus*) was used, while PVDF powder of Mw~534,000 (Sigma-Aldrich, St. Louis, MO, USA) degree of polymerization ≈ 83,338, BaTiO_3_ (BT) 99.5%, <2 μm (Sigma-Aldrich, St. Louis, MO, USA), FeSO_4_ × 7H_2_O (Acros Organics, Waltham, MA, USA), FeCl_3_ × 6H_2_O (Fisher Chemical, Waltham, MA, USA), 0.1 M NaOH, NH_3_(aq) (Sigma-Aldrich, St. Louis, MO, USA) and ethanol (Merck, Darmstadt, Germany) were used for obtaining composite films.

### 2.2. Preparation of BNC/Fe_3_O_4_

BNC was synthesized via a *K. rhaeticus* bacterial species that was isolated from a kombucha beverage [29]. Hydrogels were grown in a yeast extract−peptone−mannitol (YPM) broth at 25 °C for 7 days [16]. Afterwards, hydrogels were purified in 0.1 M NaOH at 90 °C for 2 h to remove the remaining cells and broth. The crystallinity of the obtained BNC was 76.48% [29]. The resulting BNC films were treated with diluted iron salts (FeSO_4_ × 7H_2_O + FeCl_3_ × 6H_2_O) and sonicated for 30 min. Magnetite precipitation was induced by adding NH_3_(aq) until the pH reached approximately 12. After an additional 45 min of sonication, the films were washed, dried at 40 °C for 24 h, and neutralized with distilled water.

### 2.3. Preparation of PVDF/BT/BNC/Fe_3_O_4_

**Figure 1:**
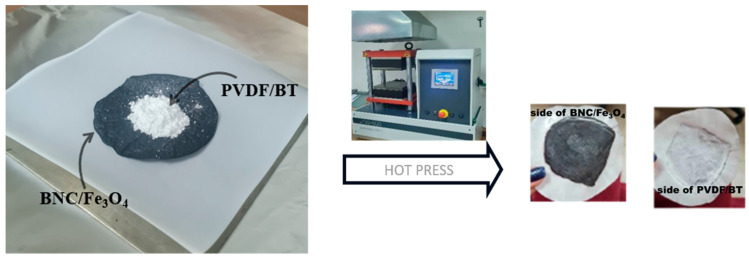
Graphical scheme of the synthesis process.

Commercial BaTiO_3_ underwent mechanical activation in a Retsch PM100 planetary mill using zirconium oxide jars and 5 mm diameter balls with 20:1 ball/powder mass ratio. The milling activation durations were 5, 10, and 20 min at a rotational speed of 400 RPM, resulting in the designation of powders as BT5, BT10, and BT20 based on milling time. The synthesis of the PVDF/BT layer involved a two-step process. Initially, 5 wt% mechanically activated BT was homogenized with PVDF in ethanol. After centrifugation and ethanol evaporation, the resulting powder mixtures were categorized as PVDF, PVDF/BT0, PVDF/BT5, PVDF/BT10, or PVDF/BT20. The fabrication of the multilayer composite comprising PVDF/BT and BNC/Fe_3_O_4_ films was achieved through hot pressing. Specifically, 1.5 g of the prepared PVDF-BT mixtures was uniformly applied to the dry BNC/Fe_3_O_4_ film. We kept the thickness of the films constant. The overall thickness of the layered composites were (0.22 ± 0.01) mm, while individual BNC/Fe_3_O_4_ layers were (0.9 ± 0.01) mm, and PVDF/BT layers had a thickness of (0.13 ± 0.01) mm [16]. Figure 1 provides a graphical depiction of the synthesis process for obtaining composite films.

### 2.4. Characterization Techniques

Scanning Electron Microscopy (SEM) and Energy-dispersive X-ray Spectroscopy (EDS) analysis were used to investigate the microstructure of synthetic films. The samples were coated with gold for 100 s at 30 mA using a Bal-tec SCD 005 Sputter coater (Schalksmühle, Germany). After preparation, the samples were imaged on a JEOL JSM-6390LV (Jeol USA Inc., Peabody, MA, USA) device equipped with software for EDS analysis, Oxford Instruments X-MaxN (High Wycombe, UK).

Fourier-transform infrared (FTIR) spectra of the samples were recorded in the transmission mode between 500 and 4000 cm^−1^ using a Thermo Scientific Nicolet iS1(Waltham, MA, USA) spectrometer with a resolution of 4 cm^−1^. The technique of attenuated total reflection (ATR) was used in this research. The spectra were recorded at room temperature.

Water vapor permeability (WVP) of the film samples was determined gravimetrically according to the ASTM E96-95 [40]. To this end, the samples were sealed to the Payne permeability cups (5100, Elcometer, Manchester, UK) containing 5 mL of distilled water with no direct contact between the films and water. A cup sealed with aluminum foil and filled with 5 mL of distilled water was tested as a control to evaluate the water loss through the sealing. The cups were stored in a desiccator containing dried silica gel on the bottom at 25 °C and weighed periodically. The WVP was calculated by Equation (1):

(1) $$WVP(g/Pa\cdot s\cdot m)=\frac{WVTR\times L}{∆P}$$

where WVTR is the water vapor transmission rate determined from the permeation slope of a weight loss in a function of time per unit of exposed film area (g/s∙m^2^), L is the film thickness (m), and ΔP is the vapor partial pressure difference between the two sides of the film (Pa).

The mechanical properties were tested using an EZ Test Table-Top Universal Testing Instruments Shimadzu cap (Kyoto, Japan). 500 N, at a 2 mm/min strain rate. The uniaxial tensile test was conducted at room temperature, and the samples were prepared by cutting them into strips with dimensions of 20 mm × 60 mm. Measurements were performed in triplicate, and ANOVA (Analysis of Variance) One-Way was used for all statistical calculations presented in the results. Considering the limited amount of synthesized films, the mechanical properties (tensile test) were measured closest to the ISO 527-3:2018 [41].

## 3. Results and Discussion

### 3.1. SEM-EDS

**Figure 2:**
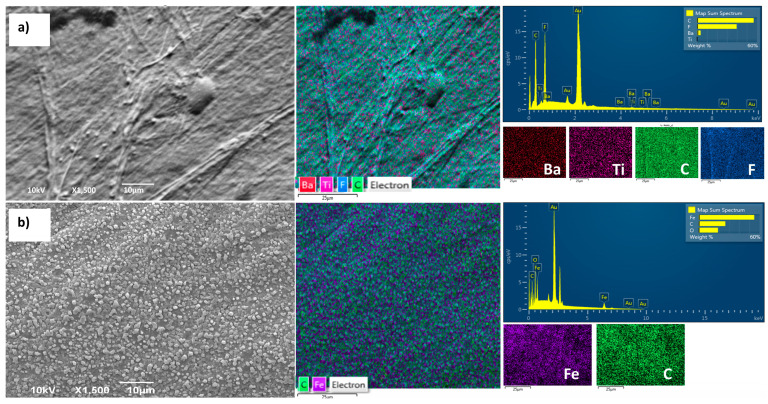
SEM and EDS of the sample PVDF/BT0/BNC/Fe_3_O_4_; (**a**) PVDF/BT0 surface side; (**b**) BNC/Fe_3_O_4_ surface side.

The compatibility of all layers in composite films is of paramount importance, as it directly affects the permeability of water vapor and other gases. In the previous research, detailed cross-sections of all samples were presented to gain insight into the achieved contact between layers [16]. To provide a comprehensive view and microscopic insight into the samples, the current study introduces SEM-EDS imaging of the surface appearance. Due to the relatively similar appearance of all synthesized composite films, the PVDF/BT0/BNC/Fe_3_O_4_ film is presented as being representative. The SEM-EDS images in Figure 2a,b show the surface morphologies of the composite film. Figure 2a illustrates the PVDF/BT side of the film, while, under Figure 2b, the BNC/Fe_3_O_4_ side of the film is shown.

Figure 2a illustrates the microstructure predominantly exhibiting the characteristics of pure PVDF, where the morphology of the pure PVDF layer depicts a dense and smooth surface, without voids, with regular folding lines appearing. Grainy aggregates of BT particles are observed, relatively homogeneously distributed throughout the PVDF film layer. The additional images of the polymer films with lower magnification are provided in the Supplementary Materials to further prove BT agglomeration.

The micrograph of the BNC/Fe_3_O_4_ layer (Figure 2b) reveals a uniform distribution of Fe_3_O_4_ particles within the BNC network. Considering that the BNC structure is highly porous (with a large number of nanopores), this is the main reason for the facilitated penetration and establishment of strong interactions with the BNC. Additional high-magnification images of the uniform distribution of Fe_3_O_4_ in the BNC network are provided in the Supplementary Materials.

### 3.2. FTIR

**Figure 3:**
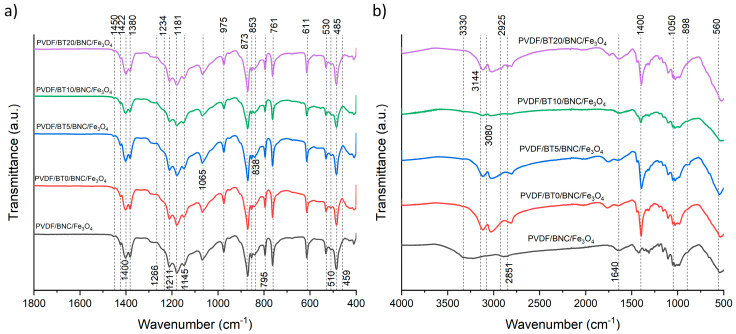
FTIR spectra of the samples. (**a**) Display of composite films from the PVDF/BT side and (**b**) the composite films from the BNC/Fe_3_O_4_ side.

The spectra of composite films collected from the PVDF/BT side are shown in Figure 3a. The appearance of the 1422 cm^−1^ peak from BT nanoparticles represents O–H in-plane deformation vibration from water molecules or the C=O band stretching vibration from CO_3_^−2^ [42]. The characteristic absorption bands of the polyvinylidene fluoride (PVDF) are observed at 838 cm^−1^, which is attributed to C-C-C stretching vibrations, while the peaks at approximately 870 cm^−1^ and 1188 cm^−1^ correspond to specific C-F vibrations [43,44]. The FT-IR technique has been used for the identification of different crystalline phases present in PVDF. Characteristic absorbance bands of PVDF observed at 485 cm^−1^, 530 cm^−1^, 611 cm^−1^, 761 cm^−1^, 766 cm^−1^ and 795 cm^−1^ are correlated to the trans-gauche TGTG’ conformation of PVDF α-phase [45], while characteristic absorption bands suggesting the presence of the β phase are located at wavelengths 510 cm^−1^, 838 cm^−1^, 873 cm^−1^ and 1234 cm^−1^ [46,47]. The bands at 510 cm^−1^, 838 cm^−1^, 873 cm^−1^, and 1234 cm^−1^ that appeared in each composite film signify the formation of the β-phase (all-trans TTTT conformation) [45,48]. Structurally and spectroscopically, the β- and γ-phases exhibit bands appear at same positions, 510 cm^−1^ and 838 cm^−1^, posing a challenge for distinguishing them. However, these two phases can be distinguished by examining the bands around 1275 and 1234 cm^−1^ to identify the presence of the β- and γ-phases [49]. On the presented spectra of all composite films, there is no clear peak at 1275 cm^−1^, exclusively indicating these bands can be ascribed to the β phase rather than γ, while the peak indicating the presence of the electroactive γ phase appeared only as shoulders at 1234 cm^−1^ [46]. Hence, all spectra of the samples exhibited the presence of both dominant α- and β- phases, where the presence of the β- phase is confirmed by peaks at 510 cm^−1^, 838 cm^−1^, and 873 cm^−1^. The mentioned phase composition is the result of the interaction of BT (nanoparticles act as nucleation agent), as well as the hot-press synthesis process [50]. Different activation times of BT do not affect the phase composition.

Figure 3b shows the BNC/Fe_3_O_4_ side of the multilayer composites. In the literature, as well as in our previous research [15,51], it was found that the characteristic peaks for the BNC/Fe_3_O_4_ sample were primarily the stretching vibration modes of the O-H group detected at a wavenumber of approximately 3340 cm^−1^, with the stretching vibration mode of the C-H group detected at a wavenumber of approximately 2900 cm^−1^. These two wave numbers with 1640 cm^−1^ for H-O-H bonding of absorbed water, 1430 cm^−1^ for bending of the C-H group, 1372 cm^−1^ for bending of -OH group, 1111 cm^−1^ for C-O-C stretching, 1060 cm^−1^ for C-O-C pyranose ring skeletal vibration, and 898 cm^−1^ for glycosidic linkage in glucose polymers, are characteristic of BNC. Furthermore, peaks at wavenumbers 580 cm^−1^ (O-Fe-O vibration), 1090 cm^−1^ (Fe-O vibration), and 1380 cm^−1^ (Fe-O vibration) serve as indicators of the presence of Fe_3_O_4_. Also, the presence of the cellulose component is confirmed by a cluster of peaks ranging from 1000 cm^−1^ to 1500 cm^−1^ [14].

The characteristic wavelengths at which BNC/Fe_3_O_4_ is detected are observed, but they have significantly lower intensity, and some peaks are shifted. It is assumed that this is due to the influence of other components that are located below the BNC/Fe_3_O_4_ layer. The stretching vibration mode of the OH- group is located at about 3330 cm^−1^, and it has a different character in the PVDF/BNC/Fe_3_O_4_ sample compared to all other samples that contain BT. At the wavenumber of 2925 cm^−1^, a stretching vibration mode of a C-H group may indicate the presence of amorphous cellulose [51,52]. This peak is not present exclusively in the PVDF/BNC/Fe_3_O_4_ sample, which does not contain BT.

The evidence of the binding of cellulose nanoparticles with Fe_3_O_4_ is the reduction in hydrogen bonds, resulting in a peak shift and detection at the wavelength of 1640 cm^−1^, which is not the case with pure BNC, where the peak is detected at higher wavelengths of around 1660 cm^−1^.

Also, in Figure 3b, the appearance of a peak at 3080 cm⁻^1^ is observed in samples with BT (PVDF/BT0/BNC/Fe_3_O_4_, PVDF/BT5/BNC/Fe_3_O_4_, PVDF/BT10/BNC/Fe_3_O_4_, PVDF/BT20/BNC/Fe_3_O_4_), indicating stretching vibration modes of Ti-OH and Ba-OH [53], which are not present in sample PVDF/BNC/Fe_3_O_4_.

### 3.3. Water Vapor Permeability

In Table 1, WVP values are presented, indicating the water vapor permeability through the tested samples, and corresponding WVTR (Water Vapor Transmission Rate) values are also included. The lower the WVP value, the better the barrier of the material against water vapor. Among the tested samples, the PVDF/BT20/BNC/Fe_3_O_4_ film exhibited the best barrier properties against water vapor (2.48 × 10^−12^ g/Pa∙s∙m).

When considering WVTR, the water vapor transmission rate is highest through samples BNC and BNC/Fe_3_O_4_, while passing through sample PVDF/BT20/BNC/Fe_3_O_4_ is the slowest, in line with WVP. Regarding samples BNC and BNC/Fe_3_O_4_, we can say that they exhibited the expected lowest barrier to the passage of water vapor, as BNC is extremely hydrophilic.

Through the literature research, a WVP value for commercial PVDF films of around 1.022 × 10^−11^ g/Pa⋅s⋅m [32] has been determined, which is comparable to our values for samples PVDF/BT0/BNC/Fe_3_O_4_ (9.07 × 10^−11^ g/Pa⋅s⋅m) and PVDF/BT5/BNC/Fe_3_O_4_ (2.63 × 10^−11^ g/Pa⋅s⋅m). Therefore, it can be assumed that, in the case of the previously mentioned samples, all added fillers (BNC, Fe_3_O_4_, and powders BT0 and BT5) do not have a significant contribution in terms of the barrier.

When it comes to sample PVDF/BT10/BNC/Fe_3_O_4_, a deviation and higher water vapor permeability compared to the previously mentioned samples was measured, finding a value of 1.40 × 10^−10^ g/Pa⋅s⋅m. Analyzing the previous study [16], a certain percentage of delamination between the layers of the PVDF/BT10/BNC/Fe_3_O_4_ sample is observed. This delamination can explain the increased water vapor permeability in the sample.

Considering the values of WVP as well as WVTR, among all tested formulations, the composite film with mechanically activated BT for 20 min exhibits the best barrier properties against water vapor. Incorporated BT 20 powder imparts superior barrier characteristics to sample PVDF/BT20/BNC/Fe_3_O_4_. Heavy deformations and the formation of defective structures, arising from the mechanical activation of BT20 powder particles, enhance the incorporation of BT into the polymer matrix, resulting in a structure that provides the highest resistance to the passage of water vapor [54].

It is interesting that, when we compare the WVP values of the obtained composite films with the WVP value of the widely used commercial PVC film (72.5 × 10^−10^ g/Pa∙s∙m) [55,56], it is clear that the samples examined in this study have significantly lower values, especially sample PVDF/BT20/BNC/Fe_3_O_4_.

### 3.4. Tensile Test

**Figure 4:**
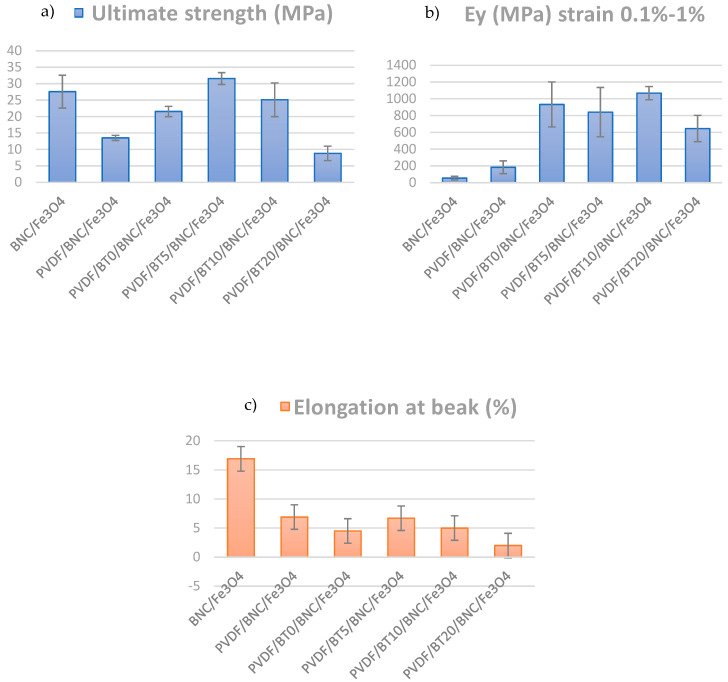
Mechanical properties of the composite films: (**a**) ultimate tensile strength and, (**b**) Young’s modulus and (**c**) elongation at break.

The characterization of the mechanical attributes of PVDF composite films holds significant importance in the context of practical applications. The mechanical properties of composite films were examined using a tensile unaxial tester, in triplicate, and are presented in Figure 4.

Mechanical properties of composite PVDF films, such as ultimate tensile strength (σ), elongation at break, and Young’s modulus (E), are calculated and summarized in Figure 4. Ultimate tensile strength represents the maximum stress that a material can endure before experiencing failure. On the other hand, Young’s modulus, also referred to as the elastic modulus, is a mechanical characteristic of linear elastic solid materials. It establishes the correlation between stress (force per unit area) and strain (proportional deformation) within the material [57]. These results were obtained by calculating the “mean of the sum” values. Standard deviations, which quantify the variation in data, are also presented in Figure 4. In comparing values of the composite strengths of samples having different activation times of barium titanate, it is observed that the highest tensile strength is exhibited by the PVDF/BT5/BNC/Fe_3_O_4_ sample, reaching 31.55 MPa. With an increase in the activation time of BT to 10 min, the value slightly decreases to 25.11 MPa, while an activation time of 20 min shows a significant drop, resulting in the lowest tensile strength value of 8.81 MPa. In comparison to the PVDF composite film without BT, the increases in ultimate strength are 69% for PVDF/BT0/BNC/Fe_3_O_4_, a substantial 139% for PVDF/BT5/BNC/Fe_3_O_4_, 92% for PVDF/BT10/BNC/Fe_3_O_4_, while, for PVDF/BT20/BNC/Fe_3_O_4,_ we have a decrease of 38%.

The mean values of Young’s modulus of elasticity presented in Figure 4b indicate that the addition of the PVDF/BT layer increases Young’s modulus values. This leads to the establishment of the mechanism of mechanical reinforcement of the polymeric composite with filler particles [58,59]. The sample with the BT activated for 10 min has the highest modulus value of 1068 MPa; meanwhile, the sample with the BT activated for 20 min yielded a modulus value of 645 MPa, which is even lower than the PVDF/BT0/BNC/Fe_3_O_4_ sample. Additionally, from Figure 4b, we observe high values of standard deviations (significant variability in the results), particularly in the case of the PVDF/BT10/BNC/Fe_3_O_4_ sample and the BNC/Fe_3_O_4_ sample. The data exhibit wide dispersion around the mean values.

In the analysis of the elongation at the break parameter, depicted in Figure 4c, significant differences were observed among individual films. For instance, the BNC/Fe_3_O_4_ sample exhibits the highest elongation value of 16.9%, indicating its capability to undergo substantial deformation before failure. Upon the addition of PVDF, the elongation at break decreases to 6.9%, and further introduction of BT leads to a slight decrease, with the PVDF/BT0/BNC/Fe_3_O_4_ sample reaching 4.5%, PVDF/BT5/BNC/Fe_3_O_4_ at 6.7% and PVDF/BT10/BNC/Fe_3_O_4_ at 5%. In contrast, the PVDF/BT20/BNC/Fe_3_O_4_ sample shows the lowest elongation value compared to the other samples, measuring only 2.0%. This can be explained by the fact that the activation time of 20 min for BT leads to more heavy deformations of the powder particles themselves, as well as agglomerates. This deformative process serves as a stress concentrator, establishing sites prone to crack growth [54]. Also, the fillers act as physical barriers that impede the movement of polymer chains, resulting in a brittle fracture [60].

**Figure 5:**
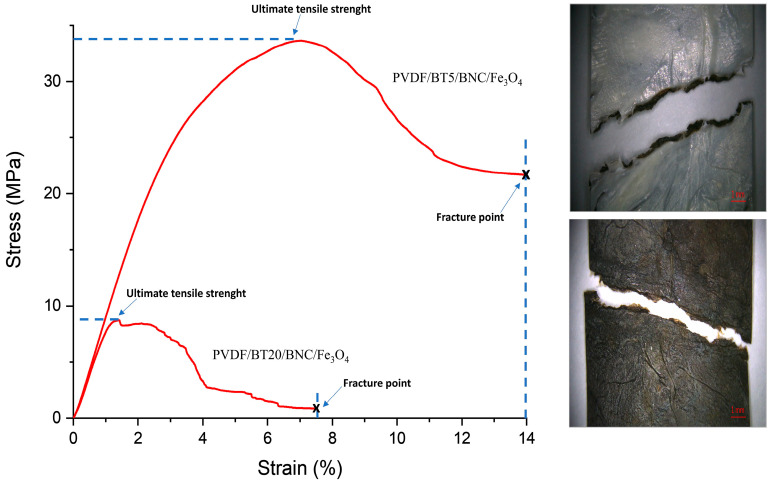
Stress versus strain comparing films of PVDF/BT5/BNC/Fe_3_O_4_ and PVDF/BT20/BNC/Fe_3_O_4_; Microscopic view of PVDF/BT20/BNC/Fe_3_O_4_ fracture.

The graph in Figure 5 illustrates the correlation between stress and strain. Based on this diagram, another important mechanical property of materials, i.e., toughness, can be assessed. Toughness is the ability of a material to absorb energy up to fracture [61]. It is calculated by integration as the area under the stress–strain curve. The larger the area, the greater the material’s toughness. In Figure 5, a comparison of the films PVDF/BT5/BNC/Fe_3_O_4_ and PVDF/BT20/BNC/Fe_3_O_4_ is shown. The PVDF/BT5/BNC/Fe_3_O_4_ film is depicted as the film with the largest stress–strain curve and a toughness modulus (Mt) of 2611.41 KJ/m^3^, thus exhibiting the highest toughness. On the other hand, composite film PVDF/BT20/BNC/Fe_3_O_4_ (Mt 285.38 KJ/m^3^) absorbs the least energy before the fracture of the film itself, so its integrated area is the smallest.

The obtained results can be explained by the presence of a dual system in the composite film where each system interacts with the other. Considering the layer of BNC with Fe_3_O_4_, it can be assumed that the embedded magnetite particles in the BNC fiber network increase the system’s friction and hinder the extraction of BNC fibers during deformation. On the other hand, the PVDF and BT system provides an additional barrier for flexible deformation. The mechanically activated BT particles facilitate interaction with PVDF due to the increased surface energy of such particles, thereby increasing the resistance to tearing over time as the mechanical activation of particles increases (BT particle−PVDF chain interactions hinder slippage). The length of mechanical activation relatively insignificantly affects the change in modulus until the critical activation time. The critical activation time that yielded the lowest results in all tested parameters, characterizing the film as the most brittle, is the activation time of 20 min. A mechanical activation length of 20 min is not suitable; it leads to particle deformation and agglomeration, resulting in a very brittle and inflexible material, which is not conducive to packaging applications.

In the images provided alongside Figure 5, there is a microscopic view of the fracture of film PVDF/BT20/BNC/Fe_3_O_4_. The film showing the white surface represents the PVDF/BT side, while the image displaying the torn film with a black surface represents the BNC/Fe_3_O_4_ surface. In the image with the PVDF/BT layer, the fracture site of both layers is visible, including the pulled-out regions of the BNC/Fe_3_O_4_ layer.

## 4. Conclusions

The study aimed to present the structural, morphological, barrier, and mechanical properties of composite films composed of PVDF/BaTiO_3_ and Fe_3_O_4_-modified bacterial nanocellulose (BNC). The layers were bonded using the hot press method. Surface SEM-EDS analysis of the films revealed that the PVDF/BT side of the film represents a smooth surface with a small number of agglomerated BaTiO_3_ particles. Meanwhile, the BNC/Fe_3_O_4_ side represents a rough, uneven surface where the Fe_3_O_4_ particles are uniformly distributed in the BNC. FTIR ATR analysis showed the preferential enhancement of beta phase peaks in all samples with BT at 510 cm^−1^, 838 cm^−1^, 873 cm^−1^ and 1234 cm^−1^ bands. Different durations of mechanical activation of BT in the samples did not affect the phase change. When it comes to barrier properties, the sample PVDF/BT20/BNC/Fe_3_O_4_ exhibited the best impermeability to water vapor. When it comes to stretching upon tearing, composite materials show a decrease in stretching with an increase in BT activation time. The highest stretching of 16.9% was exhibited by the sample BNC/Fe_3_O_4_, while the lowest stretching of 2.0% was recorded in the sample PVDF/BT20/BNC/Fe_3_O_4_; however, that same sample exhibited the least elongation upon the tensile test, indicating the highest stiffness among all samples. The sample PVDF/BT5/BNC/Fe_3_O_4_ exhibits the highest tensile strength as well as the highest toughness. When it comes to the modulus of elasticity, the sample with BT activated for 10 min (PVDF/BT10/BNC/Fe_3_O_4_) has the highest modulus value of 1068 MPa. In light of the potential application of this material as an intelligent and smart packaging material, and taking into account all presented results, the best candidate is PVDF/BT5/BNC/Fe_3_O_4_. Further investigation and development of this material will reveal other favorable properties for this application.

## Figures and Tables

**Table 1:** Water vapor permeability and transmission rate through the tested films.

Sample	WVP (g/Pa∙s∙m)	WVTR (g/h∙m^2^)
BNC	6.45 × 10^−11^	34.6750
BNC/Fe_3_O_4_	9.41 × 10^−10^	39.8479
PVDF/BNC/Fe_3_O_4_	1.30 × 10^−10^	4.4642
PVDF/BT0/BNC/Fe_3_O_4_	9.07 × 10^−11^	2.6889
PVDF/BT5/BNC/Fe_3_O_4_	2.63 × 10^−11^	0.6245
PVDF/BT10/BNC/Fe_3_O_4_	1.40 × 10^−10^	4.5896
PVDF/BT20/BNC/Fe_3_O_4_	2.48 × 10^−12^	0.0903

## Data Availability

The original contributions presented in the study are included in the article/Supplementary Material. Further inquiries can be directed to the corresponding author/s.

## Supplementary Materials

The following supporting information can be downloaded at: https://www.mdpi.com/article/10.3390/polym16081033/s1, Figure S1: SEM of the sample PVDF/BT0/BNC/Fe_3_O_4_, Fe_3_O_4_/BNC surface side (×4500, 10 kV), Figure S2: SEM of the sample PVDF/BT0/BNC/Fe_3_O_4_, PVDF/BT surface side (×450, 10 kV).
