# Supplementary material for: Structural, Mechanical, and Barrier Properties of the Polyvinylidene Fluoride-Bacterial Nanocellulose-Based Hybrid Composite
Source: Polymers (Basel). 2024 Apr 10;16(8):1033. doi: 10.3390/polym16081033 (PMC11054639; doi:10.3390/polym16081033)

Figure S1: SEM of the sample PVDF/BT0/BNC/Fe_3O_4, BNC/Fe_3O_4 surface side (x4500,10kV)

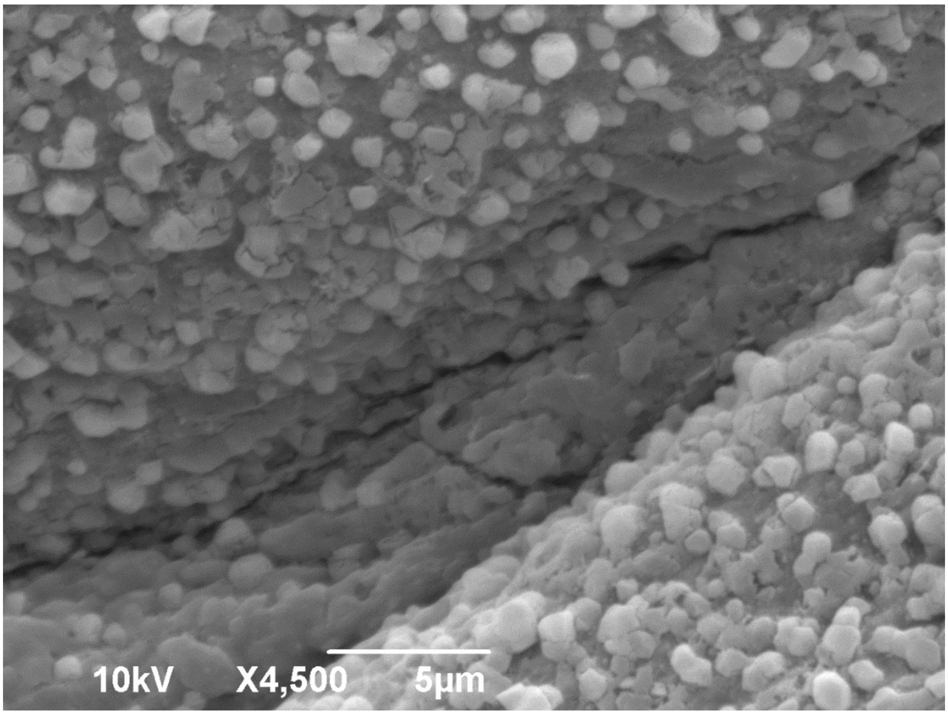

Figure S2: SEM of the sample PVDF/BT0/BNC/Fe_3O_4, PVDF/BT surface side (x450,10kV)

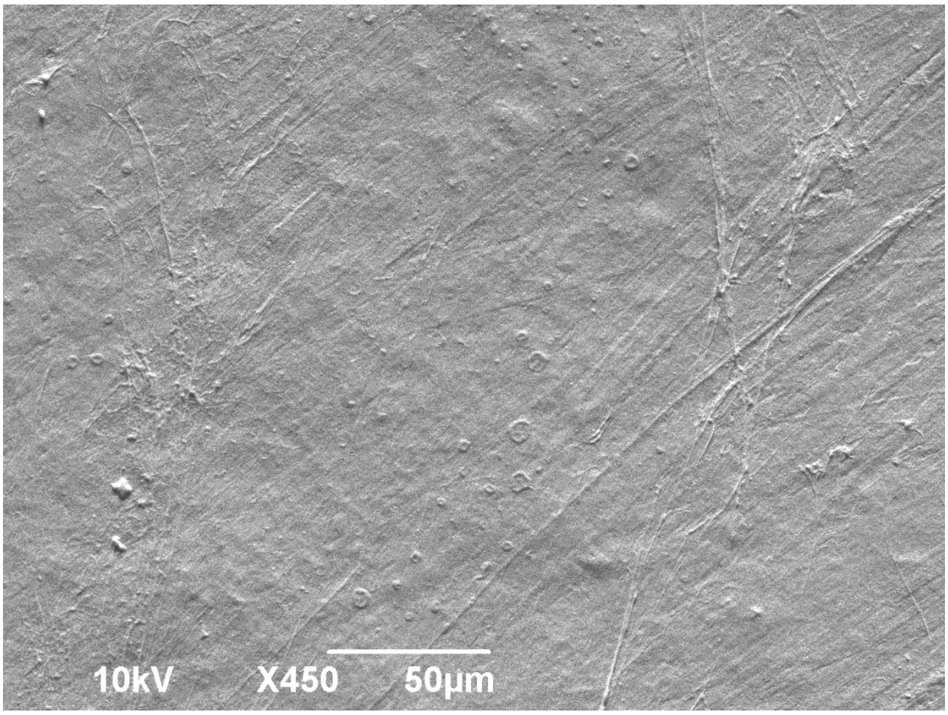

Supplement: Supplementary file 1 [file polymers-16-01033-s001.zip › polymers-2927646-supplementary.pdf]
